# Modulation of Circulating Macrophage Migration Inhibitory Factor in the Elderly

**DOI:** 10.1155/2014/582586

**Published:** 2014-07-08

**Authors:** Christos Rammos, Ulrike B. Hendgen-Cotta, Julia Pohl, Matthias Totzeck, Peter Luedike, Volker T. Schulze, Malte Kelm, Tienush Rassaf

**Affiliations:** Division of Cardiology, Pulmonology and Vascular Medicine, Medical Faculty, University Hospital Düsseldorf, Moorenstraß 5, 40225 Düsseldorf, Germany

## Abstract

Aging increases the risk for cardiovascular morbidity and mortality. Chronic low-grade inflammation deteriorates vascular function, increases age-related vascular stiffness, and affects hemodynamics. The proinflammatory cytokine macrophage migration inhibitory factor (MIF) is a major mediator of atherosclerosis. Plasma MIF levels are associated with arterial stiffness, a hallmark of vascular aging. Preclinical studies show that blockade of MIF leads to atherosclerotic plaque regression. Nutritional approaches provide opportunities to counteract age-related inflammation. Following a chronic dietary supplementation with the micronutrient nitrate has been demonstrated to improve vascular stiffness. Whether dietary nitrate affects circulating MIF levels is not known. In a randomized placebo-controlled, double-blinded study, elderly subjects received a dietary nitrate supplementation for 4 weeks. Dietary nitrate led to a decrease in plasma MIF levels in the elderly and to an improvement in vascular functions. This was associated with a reduction in central systolic blood pressure. Our data show that supplementation with dietary nitrate is associated with a reduction of circulating MIF levels along with an improvement in vascular function. This supports the concept of dietary approaches to modulate age-related changes of vascular functions.

## 1. Introduction 

The physiological aging process leads to deterioration of vascular integrity and homeostasis and these alterations have a relevant effect on cardiovascular disease [1]. A recent study demonstrated that vascular stiffness in the elderly precedes and contributes to incident hypertension with an increase in cardiovascular morbidity and mortality [2]. Dysfunctional endothelium and increased vascular stiffness are the main features of preclinical atherosclerosis and aging blood vessels supply the environment for vascular disease progression. A potential cause of vascular dysfunction within advancing age is considered the chronic low-grade inflammation of the vessel wall [3]. The upregulation of the inflammatory response is the consequence of a remodeling of the innate and acquired immune system with a chronic inflammatory cytokine production [4, 5].

The proinflammatory cytokine macrophage migration inhibitory factor (MIF) has gained attention due to its proatherogenic properties [6]. MIF was shown to be involved in inflammatory atherosclerosis pathogenesis and is implicated in modulation of disease progression [7, 8]. Clinical evidence indicates an association of MIF plasma levels with diminished endothelial function and increased vascular stiffness in patients with established cardiovascular risk [9]. Plaque regression and a more stable plaque phenotype have been shown through MIF blockade in a preclinical setting [6]. Whether modulation of circulating plasma MIF levels is feasible has not been investigated so far.

Despite efforts in pharmacological advances and dedicated medical treatment options, the cardiovascular disease burden remains high in the elderly population [10]. A healthy lifestyle and in particular a healthy diet may support physiological and healthy aging. Nutritional interventions and appropriate diets provide options to delay or even counteract age-related inflammation [11]. Adherence to certain dietary patterns is of significant importance and has been shown to impact mortality [12]. A short-term dietary nitrate supplementation affects vascular function and acutely lowers diastolic blood pressure in young volunteers [13]. The micronutrient inorganic nitrate is abundant in leafy green vegetables and is metabolized* in vivo* to nitrite, nitric oxide, and other nitrogen oxides [14]. This is particularly relevant, as nitric oxide regulates cardiovascular homeostasis under physiological and pathological conditions [15–17]. In elderly volunteers with moderately increased cardiovascular risk, a chronic dietary nitrate supplementation reversed age-related vascular dysfunction and improved prognostic relevant outcome measures [18].

Based on these findings we sought to investigate the effect of a chronic dietary nitrate supplementation on circulating MIF levels following improved vascular functions.

## 2. Methods

### 2.1. Study Population

The study was performed in accordance with the Declaration of Helsinki, and the Heinrich-Heine University Dusseldorf Institutional Ethics Committee approved the study protocol (Clinicaltrials.gov NCT01729234). 21 elderly volunteers gave informed consent and were included in a randomized, placebo-controlled double-blind trial, as published previously [18]. One volunteer was excluded in the present analysis due to a gastrointestinal disorder at baseline. Volunteers received dietary nitrate (sodium nitrate 150 *µ*mol/kg body weight; dose is equivalent to a portion of spinach) for 4 weeks and were compared to control (sodium chloride 150 *µ*mol/kg body weight), as previously described [18]. Measurements were performed before and 1 day after the last intake of the 4-week ingestion regimen with nitrate and placebo, respectively.

Blood was drawn for clinical routine and the Institute of Clinical Chemistry and Laboratory Diagnostics, University Hospital Dusseldorf, performed all analyses unless noted otherwise.

### 2.2. MIF Plasma Levels

MIF was determined as described previously [19]. Briefly, heparinized full blood was centrifuged at 800 g for 10 min (4° Celsius). The resulting plasma aliquots were snap-frozen in liquid nitrogen and stored at −80° Celsius until further analysis. MIF levels were measured by quantitative sandwich enzyme-linked immunosorbent assay (ELISA) (Quantikine, R&D Systems, Minneapolis, USA) according to the manufacturer's protocols.

### 2.3. Central Hemodynamics

Brachial blood pressure (BP) was measured in duplicate by cuff and mercury sphygmomanometer after participants had rested in a seated position for 10 min and the average of 2 measurements was recorded. The indirect measures of central hemodynamics were obtained with the subject in a supine position by using the SphygmoCor system (AtCor Medical, Sydney, Australia) as previously described [9]. Radial arterial pressure waveforms were obtained by applanation tonometry and central arterial waveforms were generated using a validated inbuilt transfer function. Applanation tonometry has been validated to yield precise assessments of intra-arterial pressures by comparison with simultaneous invasive pressure recordings [20]. The system provided a corresponding central aortic pulse waveform from which central SBP (cSBP), central DBP, and augmentation pressure (AP) are identified.

### 2.4. Statistical Methods

Results are expressed as mean ± standard error (SEM). Differences between groups were compared using unpaired Student's two-tailed *t*-test. Within group analysis was conducted with Student's paired *t*-test. Correlations between individual parameters were calculated using univariate analyses. Results are expressed as Pearson's *r* and corresponding *P* values. *P* values of less than 0.05 were regarded statistically significant. All statistical tests were conducted using SPSS 21.0 (IBM) and Prism 5.0 (GraphPad) for Mac OS.

## 3. Results and Discussion

Aging is the key nonmodifiable cardiovascular risk factor and leads unequivocally to diverse disadvantageous alterations of the cardiovascular system with the consequence of increased cardiovascular morbidity and mortality [1]. Strategies to delay the cardiovascular aging process are needed. We included community-dwelling elderly volunteers, representative for the general population. Subjects had an increased cardiovascular risk with moderately increased heart score, elevated cholesterol levels, slightly increased body mass index, and mild systolic hypertension. No history, signs, or symptoms of cardiovascular disease were noted. Baseline characteristics are given in Table 1. The moderate increase in cardiovascular risk is in line with studies examining healthy, well-functioning men and women free of cardiovascular disease in the general population [21, 22].

A typical feature and possible key mechanism of the vascular aging process is believed to be a chronic, low-grade inflammatory status [3]. Actually, inflammation has emerged as one potential cause in the pathogenesis of major age-related diseases such as atherosclerosis, type 2 diabetes, and renal diseases [23–25].

### 3.1. Age-Related MIF Expression

**Figure 1:**
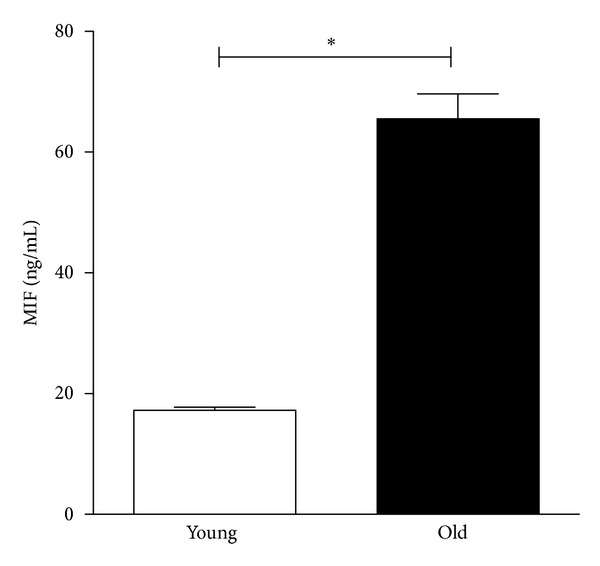
MIF plasma levels are increased in old healthy volunteers compared to young controls (∗ denotes *P* < 0.05).

The pleiotropic cytokine MIF has been attributed proinflammatory properties and is involved in atherogenic and in particular cardiovascular disease progression [8, 26, 27]. Recent reports have substantiated the inflammatory link by demonstrating that the MIF expression is linked to NF-*κ*B signaling network and that proinflammatory stimuli can activate the expression of MIF [28]. Age-related modulation was unknown until now. In the present study we determined increased MIF plasma levels in the elderly compared to young controls (65.5 ± 4.1 ng/mL versus 17.2 ± 0.5 ng/mL, *P* < 0.0001, Figure 1). Recently, we used gene expression microarray technology to determine age-related changes of the vascular transcriptome in mice. Upregulated MIF levels were shown in the old aorta compared to young, which emphasizes an age-related transcriptional regulation of MIF in vascular tissue [29].

**Figure 2:**
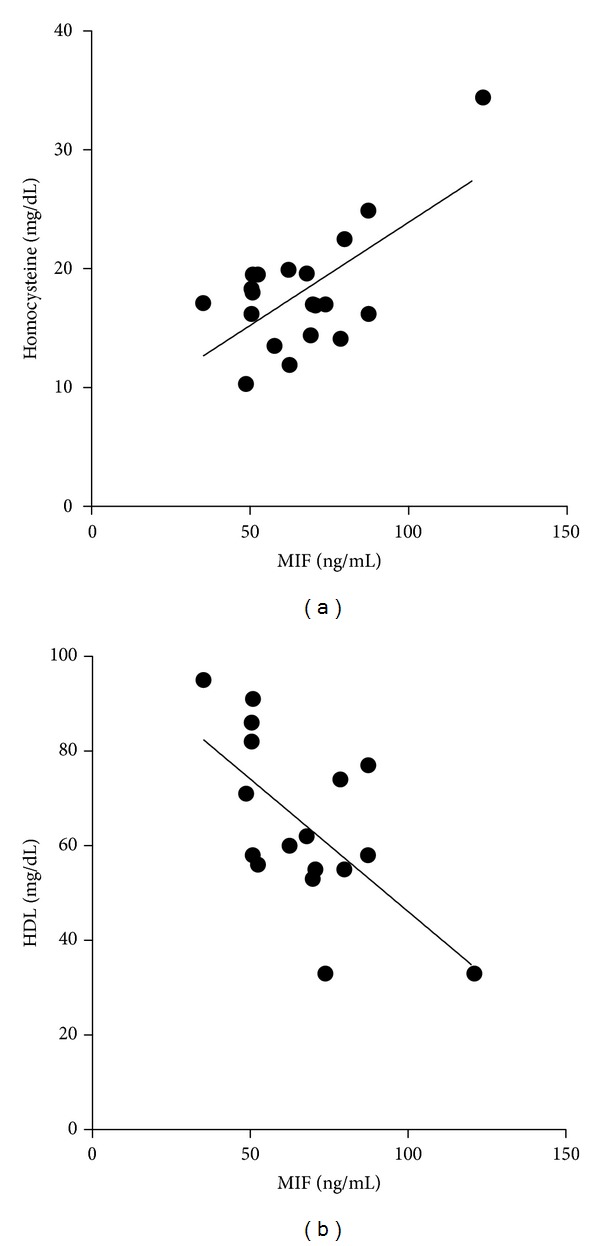
(a) MIF levels are correlated with homocysteine levels in the elderly (*r* = 0.64, *P* = 0.002). (b) MIF plasma levels are related to high-density lipoproteins (HDL, *r* = −0.64, *P* = 0.006).

Corroborating MIF's role in the age-related atherogenesis process we further found significant associations with established risk factors. Homocycteine, a recognized cardiovascular risk factor, was related to MIF in the present study (*r* = 0.64, *P* = 0.002, Figure 2(a)) [30]. Plasma high-density lipoprotein levels were associated inversely with MIF plasma levels (*r* = −0.64, *P* = 0.006). The contrariwise relation is in line with the notion that HDL has anti-inflammatory properties [31]. These results are in line with MIF's involvement in the preclinical atherosclerosis process based on low-grade inflammation [26]. Further mechanistic studies have to prove a direct link here. Importantly, experimental data suggest a beneficial effect through MIF blockade with impaired T-cell recruitment and atherosclerotic plaque regression [6, 32].

A preclinical study suggested that MIF plays a role in controlling mammalian life span. The authors observed that MIF-deficient mice lived longer than their control counterparts [33]. Thus, targeting MIF with pharmacological and nonpharmacological options seems an important matter to be addressed, with possible beneficial effects affecting health span or even longevity.

### 3.2. Dietary Nitrate Reduces MIF Levels

**Figure 3:**
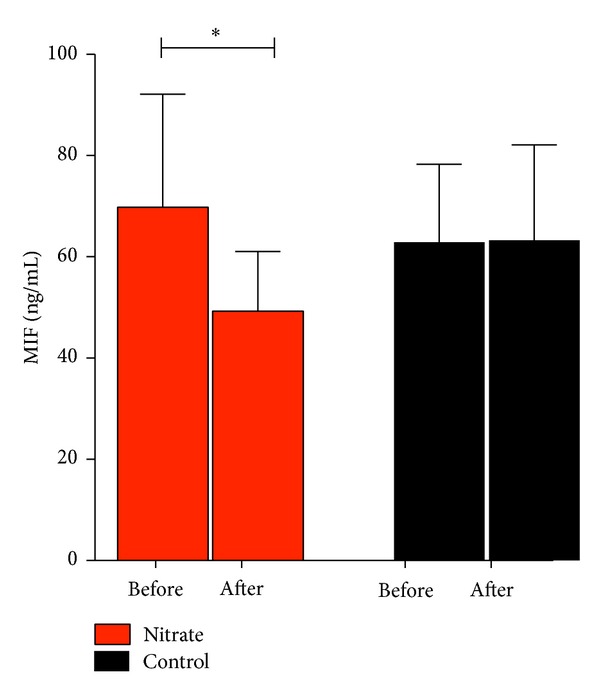
MIF plasma levels are decreased after chronic dietary nitrate supplementation in elderly volunteers (∗ denotes *P* < 0.05).

Nutritional interventions and lifestyle interventions are the cornerstone of cardiovascular disease prevention strategies. In particular, adherence to a Mediterranean dietary pattern is of great importance and has been shown to impact mortality [12]. Although there exists dispute regarding the effect of definite micro- and macronutrients on vascular functions, the groundbreaking DASH (Dietary Approaches to Stop Hypertension) trial demonstrated that certain dietary patterns influence blood pressure [34–36]. Abundant in our everyday diet and especially in leafy green vegetables is the micronutrient inorganic nitrate. Nitrate can be bioactivated via the reduction to nitrite by symbiotic bacteria in the oral cavity and is consecutively converted to nitric oxide (NO) [37–39]. A dietary nitrate supplementation has thus emerged as a possibility to enhance NO signaling and replenish NO bioavailability [14, 40]. Improved vascular remodeling was demonstrated in an animal model of hind-limb ischemia after dietary nitrate intervention [37]. In healthy volunteers ingestion of inorganic nitrate enhanced blood flow in combination with a reduction in blood pressure [41]. We showed that a dietary nitrate supplementation improves endothelial dysfunction and vascular stiffness in the elderly [18]. These outcome measures have been shown to predict cardiovascular events in old adults [42, 43]. Succeeding the reversal of age-related vascular dysfunction with accompanied increased nitrate and nitrate levels [18], we now determined reduced MIF levels following a 4-week dietary nitrate supplementation (69.8 ± 7.1 ng/mL to 49.3 ± 3.7 ng/mL, *P* < 0.05, Figure 3). No effect was observed for controls (62.9 ± 4.8 ng/mL to 63.3 ± 6.0 ng/mL, *P* > 0.05). Clearly, this study cannot provide a mechanistic link. It remains elusive whether reduced MIF levels are the consequence of dietary nitrate treatment due to increased NO bioavailability or the result of improved vascular functions. Possible interactions following a nitrate-rich diet have to be considered. MIF has previously been shown to be targeted by S-nitrosylation and to improve cardioprotection after acute ischemia and reperfusion through modulation of intrinsic oxidoreductase activity [44, 45]. Whether dietary nitrate additionally modifies MIF's function in the present study remains speculative and should be addressed in further research. Nonetheless, there exist efforts to counteract the age-related low-grade inflammation through dietary approaches [46]. In a preclinical study dietary nitrite supplementaton was shown to modulate age-related inflammatory cytokines in mice [47]. We, however, only focused on the role of dietary nitrate on MIF. Further studies are necessary to investigate effects on other cytokines.

### 3.3. Central Hemodynamics

Chronic inflammation directly influences premature atherosclerosis and arterial stiffness [48]. Vascular stiffening is considered one of the most important alterations to aged vessels. It can be measured noninvasively and is regarded as an independent predictor of cardiovascular disease events [43, 49, 50].

Increased vascular stiffness affects timing and magnitude of central pulse wave reflections, altering central hemodynamics. Consequently ventricular loading conditions and coronary blood flow are disturbed and the risk for end-organ damage is elevated [51]. This impacts on cardiac functions, limiting prognosis, and is thus implemented in recent guidelines [52].

**Figure 4:**
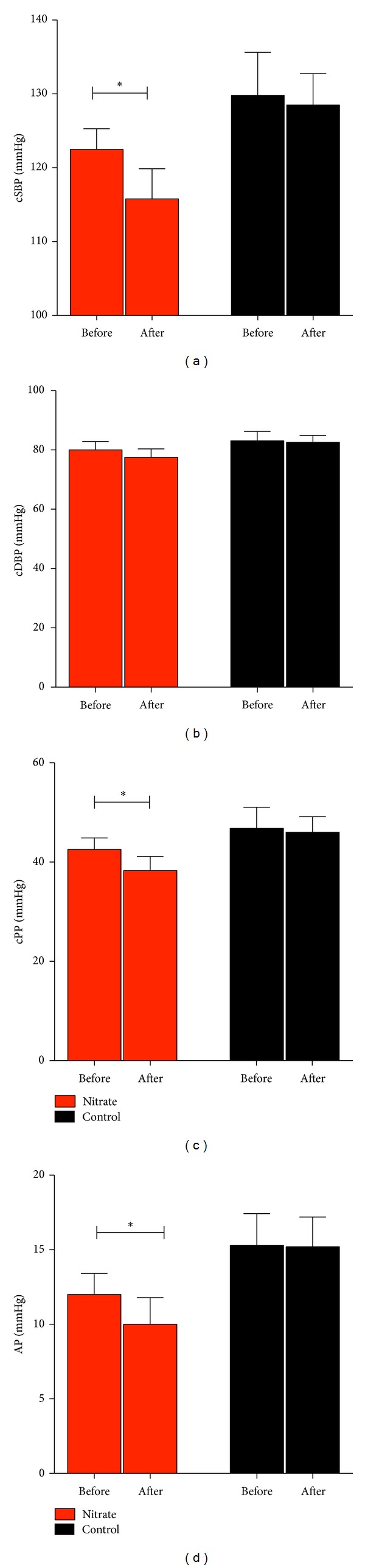
Dietary nitrate improves central hemodynamics and arterial stiffness. (a)-(b) Dietary nitrate reduced central systolic blood pressure (cSBP), while central diastolic blood pressure (cDBP) remains unaffected. (c) Central pulse pressure (cPP) was reduced following nitrate supplementation. (d) Augmentation pressure (AP) was decreased after nitrate rich diet (∗ denotes *P* < 0.05).

Improvements in peripheral vascular functions following nitrate supplementation with reduced proinflammatory MIF levels suggest an effect on central hemodynamics. Reduced central systolic blood pressure was determined in the elderly after nitrate supplementation with no effect on controls (nitrate 122.5 ± 2.8 mmHg to 115.8 ± 4.0 mmHg, *P* < 0.05, and controls 129.8 ± 5.8 mmHg to 128.5 ± 4.3 mmHg, *P* = ns, Figure 4(a)). This was accompanied by reduced central pulse pressure (cPP: nitrate 42.5 ± 2.3 mmHg to 38.3 ± 2.8 mmHg, *P* < 0.05, and controls 46.8 ± 4.2 mmHg to 46.0 ± 3.2 mmHg, *P* = ns, Figure 4(b)). No effect was observed for central diastolic blood pressure in nitrate treated elderly volunteers (*P* = ns, Figure 4(c)). Following inorganic nitrate ingestion, reduction in diastolic blood pressure has been described in healthy young volunteers before [13, 53]. We here, however, investigate elderly subjects, who show alterations in vascular integrity and function. We therefore suggest an age-dependent effect of dietary nitrate, with no effect on diastolic blood pressure. Improvement in central blood pressure after nitrate supplementation was substantiated by reduced wave reflection and stiffness indices assessed by augmentation pressure (nitrate 12.4 ± 1.6 mmHg to 9.4 ± 1.7 mmHg, *P* < 0.05, and controls 15.3 ± 2.1 mmHg to 15.2 ± 2.0 mmHg, *P* = ns, Figure 4(d)). These results are in line with studies showing attenuation of inflammation through dietary interventions and associated reductions in arterial stiffness [54]. The observed hemodynamic changes may thus implicate reduced cardiovascular risk in the elderly following dietary nitrate supplementation.

## 4. Conclusion

Our findings show that MIF levels are increased in the elderly. Following a chronic dietary nitrate supplementation, reduced MIF plasma levels are observed. Improvement in vascular functions and inflammation is substantiated by improved central hemodynamics in the elderly. This supports the concept of a dietary approach to modulate age-related vascular alterations.

## Figures and Tables

**Table 1:** Baseline patient characteristics.

	Control *n* = 10	Nitrate *n* = 10	*P* value
Age (years)	62.6 ± 1.3	63.7 ± 2	0.66
Sex (men/women)	6/4	7/4	0.87
Heart score	4.7 ± 1	4.7 ± 1	0.98
BMI (kg/m^2^)	26.2 ± 0.8	23.9 ± 1.2	0.13
Creatinine (mg/dL)	0.8 ± 0.04	0.9 ± 0.06	0.41
Hemoglobin (g/dL)	14.5 ± 0.4	14.1 ± 0.2	0.32
Triglycerides (mg/dL)	104 ± 13	126 ± 21	0.38
Cholesterol (mg/dL)	219 ± 14.1	236 ± 12.2	0.40
HDL (mg/dL)	68 ± 6	62 ± 7	0.47
LDL (mg/dL)	154 ± 9	156 ± 13	0.91
Sodium (mmol/L)	142 ± 0.6	142 ± 0.6	1.00
Potassium (mmol/L)	4.1 ± 0.1	4.2 ± 0.1	0.34
CRP (mg/dL)	<0.3	<0.3	0.33
Hba1c (%)	5.6 ± 0.1	5.8 ± 0.1	0.15

Selected demographic, clinical, and biochemical parameters. Values are expressed as mean ± SEM.
